# Ischemic Stroke: An Underestimated Complication of COVID-19

**DOI:** 10.14336/AD.2021.0209

**Published:** 2021-06-01

**Authors:** Wen Cao, Cong Zhang, Huan Wang, Qianqian Wu, Yujia Yuan, Junmin Chen, Shuo Geng, Xiangjian Zhang

**Affiliations:** ^1^Department of Neurology, Second Hospital of Hebei Medical University, Shijiazhuang, Hebei, China; ^2^Hebei Key Laboratory of Vascular Homeostasis and Hebei Collaborative Innovation Center for Cardio-cerebrovascular Disease, Shijiazhuang, Hebei, China; ^3^Department of Biological Sciences, Virginia Tech, Blacksburg, Virginia 24061, USA

**Keywords:** COVID-19, ischemic stroke, SARS-CoV-2, coagulopathy, ACE2

## Abstract

The coronavirus disease 2019 (COVID-19) has spread rapidly as a pandemic around the world. In addition to severe acute respiratory syndrome, more and more studies have focused on the complication of COVID-19, especially ischemic stroke. Here, we propose several pathophysiological processes and possible mechanisms underlying ischemic stroke after COVID-19 for early prevention and better treatment of COVID-19-related stroke.

The coronavirus disease 2019 (COVID-19) pandemic represents a challenging issue worldwide. The new coronavirus in this outbreak called severe acute respiratory syndrome coronavirus 2 (SARS-CoV-2) is a lineage of β-CoVs and identified as the seventh human-infected corona viruse. Coronaviruses (CoVs) are a subfamily of single-stranded positive-sense RNA viruses which belong to the coronavirinae family [1]. Accroding to phylogenetic clustering, CoVs are classified into α, β, γ, and δ. Among them, α-CoVs (HCoV-NL63, HCoV-229E, HCoV-HKU2, HCoV-OC43) and β-CoVs (SARS-CoV, MERS-CoV) possess the ability to infect mammal and human cells and cause respiratory diseases [2]. α-CoVs infection is mild and self-limiting, accounting for 15% to 30% common cold, whlie β-CoVs infection is responsible for severe respiratory diseases, such as SARS and MERS [3].

This outbreak of COVID-19 has the characteristics of low pathogenicity and high transmissibility compared to the SARS in 2003 and MERS in 2012 [4]. Similar to MERS and SARS, the typical symptoms of COVID-19 are various including fever, breathing difficulties, shortness of breath, cough, and fatigue. Despite the characteristic symptoms of respiratory distress, patients with COVID-19 also have neurological manifestations, especially ischemic stroke (Table 1), which has attracted considerable public attention. However, the potential mechanisms underlying COVID-19-related stroke remain unknown.

## The characteristics of COVID-19 patients with ischemic stroke

### Ischemic stroke is more likely to happen in severe cases

Studies have gradually uncovered the link between COVID-19 and ischemic stroke. Mao et al. first described that 36.4% of patients hospitalized with COVID-19 diagnosis exhibited neurological manifestations, and 4 in 88 cases (4.5%) with severe COVID-19 were affected by ischemic stroke, which was a higher frequency than that in mild cases (1 in 126 cases, 0.8%) [5]. Similarly, a higher proportion(67%) of cases with COVID-19 suffered from neurological feature in France, and this group revealed the association between disease severity and ischemic stroke incidence: 3 of 13 patients (23%) with severe COVID-19 had ischemic strokes [6].

**Table 1:** Articles on Stroke in COVID-19.

No.	Title	Type	Main points	Date	City, Country
1	Clinical Characteristics of 138 Hospitalized Patients With 2019 Novel Corona virus-infected Pneumonia in Wuhan	case series (n=138)	5.1% (7/138) of COVID19 patients have a comorbidity with cerebrovascular disease	2020.01.01-2020.02.03,	Wuhan, China
2	Neurologic Manifestation of Hospitalized Patients with Coronavirus Disease 2019 In Wuhan, china	systemic study (n=214)	78/214 patients (36.4%) had neurologic manifestations. Acute cerebrovascular disease (mainly ischemic stroke) was more common among 88 patients with severe COVID-19 than those with no severe disease (5.7% vs 0.8%).	2020.01.16-2020.02.19	Wuhan, China
3	Stroke in Patients with Sars-cov-2 Infection: Case Series	case series (n=6)	Six patients developed acute stroke during COVID-19 infection	2020.03.16-2020.04.15	Brescia or Udine, Italy
4	Acute Ischemic Stroke Complicating Common Carotid Artery Thrombosis During A Severe COVID -19 Infection	case report (n=1)	the first case of acute brain infarction due to common carotid artery thrombus in the course of a severe COVID-19 infection	2020.03.25	Toulouse, France
5	Neurologic Features in Severe Sars-cov-2 Infection	systemic study(n=64)	58 patients with severe COVID-19 infection had neurologic manifestations	2020.03.03-2020.04.03	Strasbourg, France
6	Large-vessel Stroke as A Presenting Feature Of COVID-19 In the Young	case series (n=5)	Five cases of large-vessel stroke in patients younger than 50 with COVID-19	2020.03.23-2020.04.07	New York, USA
7	Venous and Arterial Thromboembolic Complications In COVID19 Patients Admitted To An Academic Hospital In Milan, Italy	systemic study (n=388)	2.5% (9/388) COVID-19 infected patients were diagnosed ischemic stroke	2020.02.13-2020.04.10	Milan, Italy
8	Characteristics of Ischemic Stroke Associated With COVID-19	case series (n=6)	COVID-19 associated ischemic stroke is usually delayed	2020.04.01-2020.04.16	London, UK
9	COVID-19 Presenting as Stroke.	case series (n=4)	A series of 4 COVID-19 patients presented cerebrovascular accident in early stages of illness	Not mentioned	NY, USA
10	Cerebrovascular Disease In COVID-19	case report (n=1)	The first reported case of COVID-19-related cerebral infarcts including brain imaging at multiple time points and CT angiographic imaging	Not mentioned	Pennsylvania, USA
11	Stroke in A Young COVID-19 Patient	case report (n=1)	a case of large-vessel stroke after COVID-19 infection	Not mentioned	Bridgeport, USA
12	Hemorrhagic Stroke And COVID-19 Infection: Coincidence or Causality?	case report (n=1)	a case of first-time intracerebral hemorrhage in a patient with APP gene duplication	2020.09.22	Petropolis, Brazil
13	Pediatric Stroke Associated with A Sedentary Lifestyle During the Sars-cov-2 (COVID-19) Pandemic: A Case Report on A 17-year-old	case report (n=1)	a case associated with a sedentary lifestyle	2020.10.28	Los Angeles, USA
14	The Impact Of COVID-19 On Ischemic Stroke	case report (n=1)	a case of right limb weakness with COVID-19	2020.06.29	Wuhan, China
15	Hemorrhagic stroke in setting of severe COVID-19 Infection Requiring Extracorporeal Membrane Oxygenation (ECMO)	case report (n=1)	a case required ECMO support	2020.08.19	Florida, USA
16	Acute ischemic and hemorrhagic stroke in two Covid-19 Patients	case series (n=2)	two cases with coincident presentation of COVID-19 and cerebrovascular accident	2020.10.30	Italy
17	COVID-19 and stroke: casual or causal role?	case report (n=1)	a case of stroke in a 62-year-old COVID-19-positive patient, with multiple vascular risk factors.	2020.07.08	Frosinone, Italy
18	Ischemic-hemorrhagic stroke in patients with Covid-19	case report (n=2)	2 cases of patients infected with severe Covid-19 that were hospitalized in the Reanimation Unit that presented cerebrovascular symptoms and died afterwards	2020.10.06	Bizkaia, Espada
19	Complicacion Trombotica De Neumonia Grave Por COVID-19: Ictus Por Embolismo Paradojico Atipico.	case report (n=1)	a case with a central venous catheter, with a large vessel ischemic stroke, treated with mechanical thrombectomy for an atypical paradoxical embolism while in intensive care for bilateral COVID-19 pneumonia	2020.07.31	Barcelona, Espana
20	Clinical course of a 66-year-old man with an acute ischemic stroke in the setting of a COVID-19 Infection	case report (n=1)	A case with COVID-19 suffered from a right frontal cerebral infarct producing left-sided weakness and a deterioration in his speech pattern.	2020.08.28	Sutton-in-Ashfield, UK
21	COVID-19 is an independent risk factor for acute ischemic stroke	systemic study(n=123)	After adjusting for age, sex, and risk factors, COVID-19 infection had a significant independent association with acute ischemic stroke compared with control subjects (OR, 3.9; 95% CI, 1.7-8.9; P = .001)	2020.03.16-2020.04.05	New York, USA
22	Multiple embolic stroke on magnetic resonance imaging of the brain in a covid-19 case with persistent encephalopathy	case report	a case of multifocal ischemic stroke in a patient with COVID-19	2020.10.11	WV, USA
23	Characteristics and Outcomes of COVID-19 Associated Stroke: A UK Multicenter Case-control Study	systemic study(n=1470)	Cases with ischemic stroke patients (admitted during the period of COVID-19 between 9 March and 5 July 2020) were more likely than ischemic controls (patients admitted during the same time period who never had evidence of COVID-19) to occur in Asians (18.8% vs 6.7%, p<0.0002)	2020.03.07-2020.07.05	London, UK
24	Unusual Pattern of Arterial Macrothrombosis Causing Stroke in A Young Adult Recovered From COVID-19	case report (n=1)	a case with no significant past medical history who recently recovered from a mild COVID-19 infection and presented with unusual pattern of arterial macrothrombosis causing AIS	2020.10.12	Los Angeles, USA
25	Middle Cerebral Artery Ischemic Stroke and COVID-19: A Case Report	case report (n=1)	a case of a patient with SARS-CoV-2 infection and respiratory symptoms, complicated with a pro-thrombotic state involving multiple vascular territories and concomitant interleukin-6 increase	2020.09.10	Modena, Italy
26	Massive Bilateral Stroke in A COVID-19 Patient	case report (n=1)	a case of a woman with COVID-19 who suffered massive and bilateral middle cerebral artery strokes	2020.08.21	Birmingham, UK
27	Acute Ischemic and Hemorrhagic Stroke and COVID-19: Case Series	case series (n=5)	5 cases of adults with COVID-19 indicate that COVID-19 may damage blood vessels in the brain and lead to stroke	2020.10.08	Sanandaj, Iran

### Younger patients are also victims

Five cases of large-vessel stroke in patients younger than 50 years of age following SARS-CoV-2 infection have been reported in Mount Sinai Health System in New York City [7]. Increasing evidence suggests that ischemic stroke may occur after SARS-CoV-2 infection, even in young patients [8-11].

### Acute ischemic stroke is the most common type of cerebrovascular diseases after COVID-19

Recently, Nannoni et al. reviewed published articles on acute cerebrovascular diseases (CVD) after COVID-19 (December 2019-September 2020) and found that among 108,571 patients with COVID-19, 1.4% of patients suffered from acute CVD. The most common manifestation in CVD was acute ischemic stroke (87.4%) [9,12]. Ischemic stroke could be the first manifestation after SARS-CoV-2 infection [8]. The incidence of COVID-19-related hemorrhagic stroke did not differ from that of non-COVID-19-hemorrhagic stroke [13].

### COVID-19-related stroke has low incidence but poor outcome

As mentioned above, although the incidence of acute ischemic stroke was relatively low during hospitalization due to COVID-19, ranging from 1% to 6%, the mortality associated with it is substantially high, reaching as high as 38% [14,15]. Multivariate analysis demonstrated that patients with COVID-19 have more severe strokes and poorer outcomes compared with non-COVID-19 stroke [16]. Without other risk factors, ischemic stroke was an uncommon complication, exclusive of patients with a severe pulmonary injury. The presence of COVID-19 in patients who underwent EVT was an independent predictor of in-hospital mortality [14]. The presence of COVID-19 has been proved as an independent predictor of in-hospital mortality in ischemic stroke patients who underwent endovascular treatment [14].

These cases confirmed the connection between COVID-19 and ischemic stroke. Therefore, we conducted a commentary to investigate the possible pathophysiology of ischemic stroke after SARS-CoV-2 infection.

## Pathogenic factors of SARS-CoV-2

### Pathways of SARS-CoV-2 to invade the brain

The SARS-CoV-2 nucleotide sequence is 82% identical with that of human SARS-CoV and 50% identical with that of MERS-CoV [17-19]. SARS-CoV-2 has been identified in cerebrospinal fluid by polymerase chain reaction [20]. Several studies have suggested that SARS-CoV-2, like most coronaviruses, is neurotropic [21]. SARS-CoV-2 can plausibly invade the brain via several routes. First, SARS-CoV-2 binds to the angiotensin converting enzyme 2 (ACE2) receptor on the endothelial cells, which comprise the main component of the blood-brain barrier (BBB). SARS-CoV-2 transport across the vascular endothelium impairs the BBB and further enables virus invasion into the brain, where SARS-CoV-2 interacts with ACE2 on the surface of neurons, causing damage to the nervous system [22]. Second, SARS-CoV-2 passes across the BBB through adhesion to and subsequent infection of ACE2-expressing leukocytes, termed the *Trojan horse* mechanism [23]. Third, SARS-CoV-2 binds to the ACE2 receptor on the olfactory epithelium in the nasal cavity and invades the brain through the sieve plate near the olfactory bulb. This route is supported by observations of isolated anosmia and ageusia with or without respiratory symptoms [24,25].

### ACE2, the door for SARS-CoV-2 to enter the body

**Figure 1.**
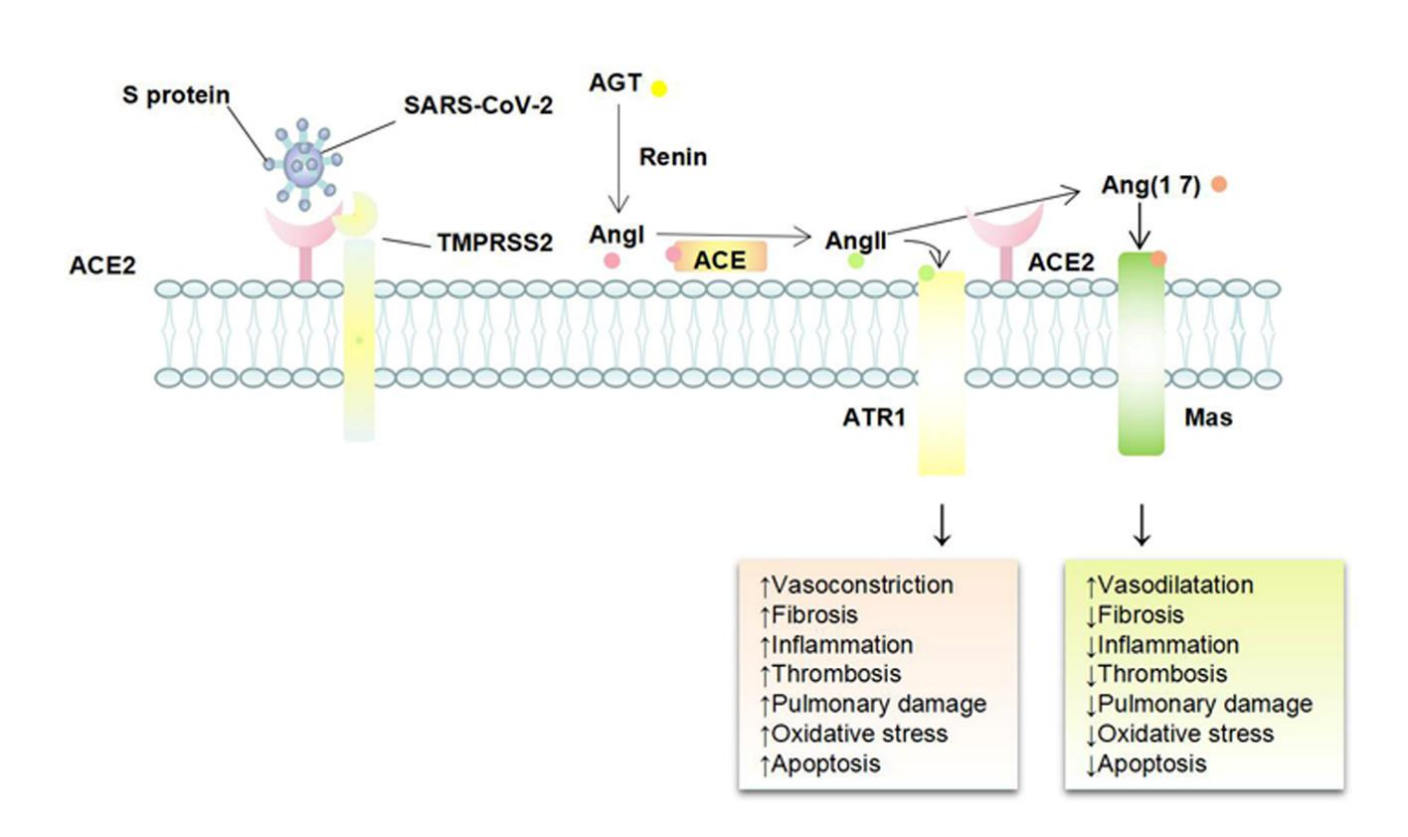
Mechanism of SARS-CoV-2 invasion and its effects on the vascular endothelium. Angiotensin converting enzyme 2 (ACE2), a dipeptidyl carboxypeptidase and a homolog of ACE, is an essential negative regulator of the renin-angiotensin system. Renin cleaves angiotensinogen (AGT) into angiotensin I (Ang I), which is hydrolyzed by ACE to angiotensin II (Ang II). Ang II has a high affinity to Ang II receptor 1 (AT1R) and plays a major physiological role in mediating vasoconstriction, neuroinflammation, oxidative stress, apoptosis, and cell proliferation, known as the classic ACE-Ang II-AT1R axis. ACE2 cleaves Ang II into Ang-(1-7), which effectively binds to the Mas receptor and counteracts adverse effects of the ACE-Ang II-AT1R axis, known as the ACE2-Ang-(1-7)-Mas receptor axis.

Recently, ACE2 was identified as the receptor for SARS-CoV-2 that caused the COVID-19 pandemic. SARS-CoV and SARS-CoV-2, which both can bind to ACE2 receptors, have 76% homology in amino acid sequence [26], but the affinity of ACE2 for SARS-CoV-2 is 10-20 times higher than that of SARS-CoV, which explains why SARS-CoV-2 is more infectious. In the brain, ACE2 is expressed in several cell types, especially in cerebral vascular endothelial cells [27]. As shown in Figure 1, Following SARS-CoV-2 binds to ACE2, the membrane receptor ACE2 is functionally removed from the outer membrane, resulting in the downregulation of ACE2 surface expression [28]. Therefore, the ACE2-Ang-(1-7)-Mas receptor axis is substantially weakened, whereas the ACE-angiotensin II (Ang II)-Ang II receptor 1 (AT1R) axis mediating vasoconstriction, neuroinflammation, oxidative stress, apoptosis, and cell proliferation functions is relatively strengthened. In addition to protecting the brain from inflammation, apoptosis, and oxidative stress, Ang-(1-7) and the Mas receptor can also reduce platelet proliferation and glycoprotein VI activation by increasing nitric oxide (NO) and prostacyclin, thereby inhibiting thrombosis [29].

Taken together, SARS-CoV-2-mediated loss of ACE2 impairs endothelial cell function, leading to the occurrence or worsening of acute ischemic stroke [30].

## COVID-19 and thrombosis

Thrombotic events include both venous thromboembolism (VTE) (deep vein thrombosis [DVT] and pulmonary embolism [PE]) and arterial thrombosis (myocardial infarction [MI], ischemic stroke, and others systemic thromboembolism). Thrombosis is commonly happened in acute infections, such as SARS [31,32] and H1N1 influenza [33,34]. The thrombotic incidence appears higher in COVID-19, even with the utilization of thromboprophylaxis [35]. A study assessed hospitalized patients with COVID-19 in a New York city health system demonstrated that thrombotic events occurred in 16.0% patients [36].

The incidence of venous thrombotic events is higher than arterial. A pooled analysis including thirty-five observational studies, showed that the pooled incidence of VTE was up to 41.9% and the pooled incidence of arterial thrombosis was 11.3% [37]. Klok et al. studied 184 ICU patients with COVID-19 and showed that PE was the most frequent thrombotic complication (81%) [38]. As to arterial thrombotic events, Modin et al found that 44 and 17 in 5119 COVID-19 patients suffered from ischemic stroke and AMI, respectively [39].

Higher incidence of thrombotic events was observed in COVID-19 patients with a severe condition [36,40]. Bilaloglu et al reported 244 in 829 ICU patients (29.4%) and 289 in 2505 non-ICU patients (11.5%) underwent a thrombotic event [36]. COVID-19 patients diagnosed with thrombotic complications were at higher risk of all-cause death (HR 5.4; 95% CI 2.4-12) [41]. Some researchers pointed out that PE was the direct cause of death behind COVID-19 [42]. In severe COVID-19 patients, the significant increase of D-dimer is a good indicator for identifying high-risk group of thrombotic complications [43], especially for PE [44]. Increased D-dimer concentrations of greater than 1.0 μg/ml effectively predict the risk of VTE in COVID-19 patients [45]. D-dimer level-guided aggressive thromboprophylaxis treatments in patients with COVID-19 has essential clinical value [46].

COVID-19 is an endothelial disease, in the end. Small fibrinous thrombi were observed in small pulmonary vessels in areas of both damaged and more preserved lung parenchyma from severe COVID-19 patients [47-49]. Two studies involved 10 and 11 decedents both found thrombosis and microangiopathy in the small vessels and capillaries of the lungs, which may lead to death [50,51]. The high incidence of thrombotic events suggests an important role of SARS-CoV-2-induced endothelial injury.

There are three triggers for thrombosis: hyper-coagulability, endothelial damage, and abnormal hemodynamics. SARS-CoV-2 infection and inflammatory reactions lead to vascular damage, hyper-coagulability, thrombin activation, platelet aggregation, as well as plaque shedding due to hemodynamic changes, thereby promoting the occurrence of ischemic stroke.

## The potential thrombosis pathogenesis behind COVID-19-related stroke

As described above, many papers have reported that patients with COVID-19 are at increased risk of thrombosis, which results in COVID-19-related stroke. We summarize several pathogeneses and propose the potential mechanisms.

### Coagulation dysfunction

Most patients with COVID-19, especially severe cases and critically ill patients, have varying degrees of coagulation dysfunction. Abnormal coagulation parameters are predictors of poor prognosis in COVID-19 patients [52]. Patients with COVID-19-related stroke have significantly higher D-dimer levels and blood viscosity than those with stroke alone [53]. A retrospective study of 191 COVID-19 patients in Wuhan demonstrated that 42% of patients with COVID-19 and 81% of deceased patients with COVID-19 had D-dimer levels higher than 1.0 μg/mL [54]. Chen et al. reported that 36 of 99 (36%) non-severe COVID-19 patients exhibited elevated D-dimer levels [55]. Tang et al. reported that non-survivors had significantly higher D-dimer and fibrin degradation products (FDP) than survivors. In the late stage of the disease, 71.4% of non-survivors and 0.6% of survivors had different degrees of disseminated intravascular coagulation, due to the excessive consumption of coagulation factors [56]. These findings suggest that coagulation disorders in COVID-19 patients are closely related to the severity of the disease.

**Figure 2.**
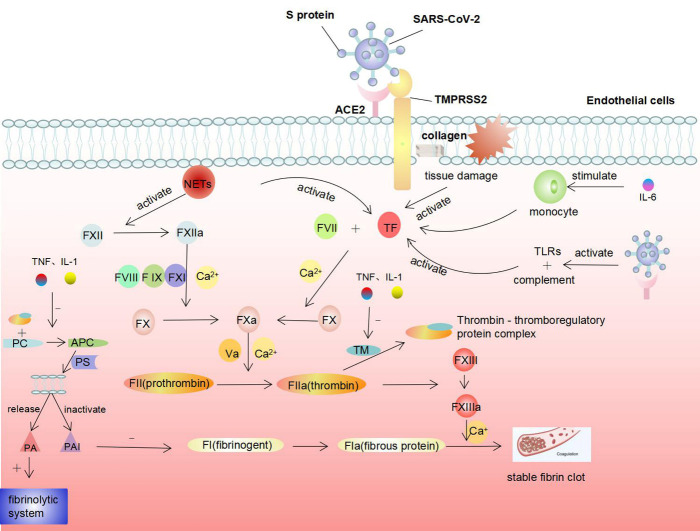
Mechanisms of coagulation dysfunction triggered by SARS-CoV-2.

Coagulation and anticoagulation systems include platelet thrombus formation, fibrin formation, and fibrinolysis. Under physiological conditions, anti-coagulation is the main factor that ensures normal blood flow. Upon vascular endothelium damage, the subendothelial collagen is exposed to blood, thereby activating the coagulation system [57], resulting in a large amount of thrombin generation and fibrin deposition, which is the key factor of hypercoagulability. We suspect five mechanisms behind SARS-CoV-2-mediated coagulopathy (Fig. 2).

#### (1)SARS-CoV-2 directly damages the endothelium and activates coagulation.

SARS-CoV-2 directly damages endothelial cells in various organs [58]. Autopsies have shown that SARS-CoV-2 virus particles are present in capillary endothelial cells, resulting in the disruption of their tight junctions, cell swelling, and loss of contact with the basement membrane, and endotheliitis [58,59]. After a vascular endothelial injury, the subendothelial collagens are exposed and release tissue factor (TF), triggering an exogenous coagulation cascade that leads to thrombin production and fibrin deposition [60].

#### (2)Excessive cytokines activate coagulation.

Excessive production and release of cytokines such as tumor necrosis factor (TNF), interleukin (IL)-1, and IL-6 are characteristic of COVID-19. TNF and IL-1 promote blood coagulation by inhibiting the expression of thromboregulatory protein (TM) and reducing the production of activated protein C (APC), thus promoting the coagulation reaction [61,62]. TM is expressed on the surface of endothelial cells and forms a thrombin-thrombomodulin complex, inhibiting the activity of thrombin. Moreover, the complex can also activate APC. APC is a strong anticoagulant that promotes the release of tissue-type plasminogen activator, thereby activating the fibrin dissolution system and inactivating tissue plasminogen activator inhibitor [62].

IL-6 stimulates fibrinogen synthesis in the liver and induces megakaryocytes to produce a large number of platelets, both of which can promote the coagulation reaction. IL-6 can also stimulate monocytes to release TF, thereby initiating the exogenous coagulation cascade [63].

#### (3)SARS-CoV-2 activates the body's immune response, leading to activation of coagulation.

Thrombosis is one of the ways in which innate immunity strives to reduce the spread of infection [64]. After infecting the human body, SARS-CoV-2 is recognized by pathogen-associated molecular patterns (PAMP) receptors like Toll-like receptors (TLRs), initiating innate immune system. In addition, the complement system is activated after SARS-CoV-2 infection. C3a and C5 cleavage products (C5a and C5b) collectively influence many aspects of coagulation, including the activation of TF, the release of von Willebrand factor (vWF) from endothelial cells, and enhancement of P-selectin exposure [65].

Peripheral lymphocyte counts (mainly CD4+ and CD8+ T cells) were consistently and significantly reduced in COVID-19 patients, especially in severe cases, suggesting that the adaptive immunity is also weakened [54,66]. The degree of lymphopenia is correlated with the severity of COVID-19 [67]. SARS-CoV-2 can directly invade lymphocytes, particularly T cells, resulting in a compromised antiviral response due to a reduction in the number of lymphocytes [66]. However, it was demonstrated that although the counts of peripheral T cells were substantially reduced, their status was highly pro-inflammatory, manifested by increased numbers of Th17 cells and high cytotoxicity of CD8 T cells [48]. Dysregulation of T cell subsets can also increase the secretion of negative hematopoietic regulators (IL-2, interferon [IFN]-γ, and TNF), leading to coagulation dysfunction.

#### (4)Neutrophils are involved in the activation of the coagulation cascade.

Stimulated by inflammatory cytokines or infectious pathogens, neutrophil extracellular traps (NETs) are released from neutrophils to capture pathogens [68]. This fibrous network provides a scaffold for platelets to adhere to and capture erythrocytes and leukocytes, leading to platelet aggregation and thrombosis. Autopsies of deceased COVID-19 patients revealed neutrophil infiltration in pulmonary capillaries [68] and the level of NETs in the blood of COVID-19 patients is increased [69]. NETs can directly activate factor XII and TF and initiate an exogenous coagulation cascade. Moreover, histones in NETs also function as ligands for platelet TLRs to promote platelet activation and thrombosis [70].

#### (5)Extramedullary megakaryocytes induce microvascular thrombosis.

Extramedullary megakaryocytes are present in the microvessels of most organs. The number of pulmonary megakaryocytes is increased by infection, impairing the respiratory system and the circulatory system [71]. Approximately 90% of lung-derived megakaryocytes are present in the pulmonary microcirculation. However, in cases of severe infection, a large proportion of megakaryocytes leave the lungs and enter the arterial circulation [72]. Megakaryocytes in the peripheral circulation can produce platelets, resulting in microvascular thrombosis in COVID-19 patients.

In summary, coagulation dysfunction increases the risk of thrombosis in COVID-19 patient.

### Role of cytokine storm in COVID-19-related stroke

The cytokine storm is characteristic in patients with the most severe forms of COVID-19. Huang et al. first noted elevated levels of pro-in?ammatory cytokines, such as IL-1, TNF-α, IFN-γ, IP-10, and MCP-1, in the serum of patients with COVID-19 [54]. Subsequently, Mehta et al. confirmed the increase of inflammatory cytokines (IL-6, IL-10, IL-2, and IFN-γ) in severe cases of COVID-19 [73]. Compared to non-ICU patients, ICU patients have higher concentrations of G-CSF, IP-10, MCP-1, TNF-α, and IL-6 [74].

The cytokine storm, also called hypercytokinemia, is the phenomenon of the aggressive release of pro-inflammatory cytokines and insufficient control of anti-inflammatory responses due to immune dysfunction [75]. Under normal circumstances, the immune system can respond to external stimuli by secreting cytokines, helping the body to overcome the attack by pathogens. Following SARS-CoV-2 invasion, the virus is first recognized by innate immune system and activates immune cells, such as macrophages and dendritic cells (DCs), which can phagocytose and hydrolyse the virus [76]. Macrophages can release TNF-α in an endocrine manner, and dendritic cells secrete IL-12 and IL-6 in a paracrine manner [77]. Once the virus escapes recognition by the innate immune system, it is recognized by cell surface RNA pattern recognition receptors and form a protein complex, which promotes the translocation of transcription factors and upregulates the expression of pro-inflammatory factors. The release of cytokines facilitates the recruitment of more immune cells and secretion of more cytokines, thereby forming a positive feedback loop to continuously amplify the inflammatory response. This positive feedback results in two outcomes: 1) Pro-inflammatory cytokines are massively produced, disrupting the balance between pro- and anti-inflammatory cytokines. 2) Due to excessive response by the immune system, the cytokines start to attack healthy tissues rather than the virus, causing a systemic inflammatory response.

**Figure 3.**
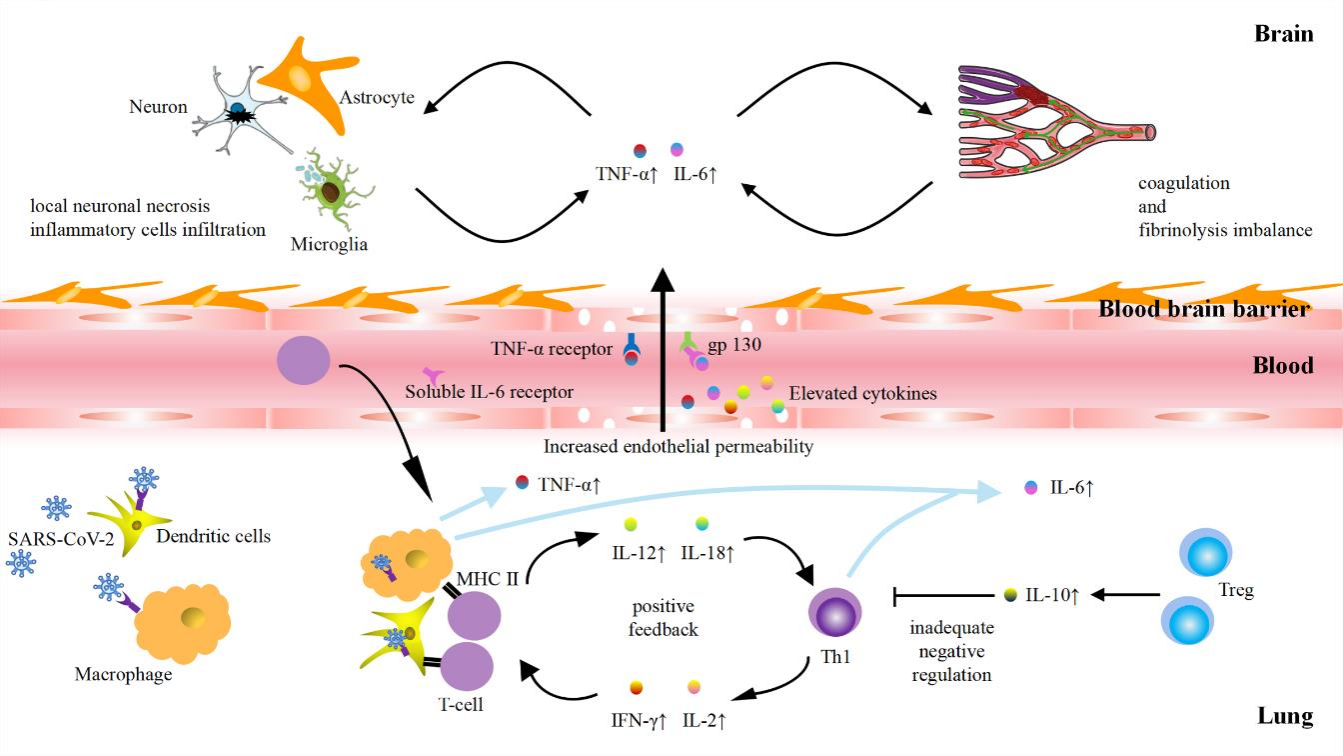
Possible mechanisms of the cytokines storm in COVID-19-associated stroke.

We hypothesize three possible mechanisms underlying cytokine storm-induced strokes after SARS-CoV-2 infection (Fig. 3). 1) Endothelial cell dysfunction leads to a disruption of the BBB. Brain endothelial cells express TNF-α and IL-6 receptors that may mediate local cell dysfunction [78], inducing the rupture of the BBB. Therefore, the integrity of the BBB is altered with increased permeability, leading to an elevated concentration of pro-inflammatory cytokines in the brain parenchyma [79]. 2) Excessive cytokines directly cause local neuronal necrosis. Enhanced permeability of the BBB enables excessive cytokine levels in the brain parenchyma, directly cause local neuronal necrosis. Necrosis may also recruit more infiltrating inflammatory cells around neurons to induce a stroke. 3) Injured endothelial cells induce coagulation and fibrinolysis imbalance. The injured endothelial cells induced by cytokines, in turn, release inflammatory factors in excess. This positive feedback promotes platelet activation and fibrinogen deposition, as well as inhibit fibrinolysis and thrombomodulin activity, leading to the imbalance of coagulation and fibrinolysis. The endothelial cells transform from an anti- to a procoagulant state, thereby facilitating thrombosis.

### Antiphospholipid antibodies

Antiphospholipid antibodies (aPLs) mainly include anticardiolipin antibodies (aCL), anti-β2GPi antibodies (anti-β2GPi), and lupus anticoagulant (LAC). Harzallah et al. reported that 25 of 56 (45%) of patients with COVID-19 were LAC-positive, and 5 (10%) of the patients were positive for aCL or anti-β2GPi antibodies [80]. In Wuhan, China, Zhang and colleagues first described that three COVID-19 patients with ischemic stroke were positive for aCL and anti-β2GPi antibodies [81]. Beyrouti et al. reported that five COVID-19 patients with ischemic stroke were LAC-positive [82]. These cases aroused our curiosity regarding the relationship between aPL and COVID-19-related stroke.

Among aPLs, LAC, the complex of aPL and plasma proteins (mainly β2-glycoproteins), is the greatest risk factor for arterial and venous thrombosis [83] and β2GPi is the main binding cofactor for these antibodies [84]. Patients positive for LAC, aCL, and anti-β2GPi antibodies, called triple-positive patients, have incidence rates for VTE of 9.8% (after 2 years) and 37.1% (after 10 years) during the follow-up period [85].

**Figure 4.**
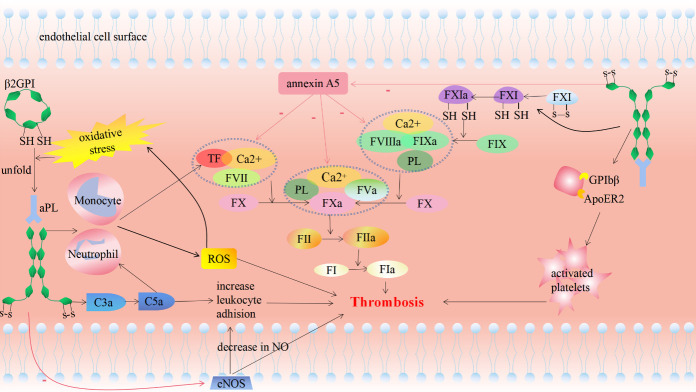
Pathophysiological mechanisms of antiphospholipid antibodies induced thrombogenesis.

The pathogenesis of aPL behind COVID-19-related stroke is multifaceted (Fig. 4). 1) Increased oxidative stress: Autoantibodies can disrupt mitochondrial function of monocytes and neutrophils, resulting in excessive reactive oxygen species (ROS) release in patients [86,87]. Following ROS stimulation, free thiols of β2GPi form disulfide bonds, and the ring conformation of β2GPi unfolds, exposing the normally shielded epitopes, thereby inducing autoantibody formation [88]. Thus, a positive feedback is formed to further promote oxidative stress. Increased levels of oxidative stress mediate damage to cell structures and serve as a trigger for ischemic stroke [89]. 2) Changes in coagulation factors: aPLs upregulate the release of TF, which is the key promoters of the exogenous coagulation cascade, from monocytes [86], neutrophils [87] and endothelial cells [90]. Furthermore, aPLs increase the level of coagulation factor Ⅺ containing free thiols (reduced factor Ⅺ), which is more prone to be activated by thrombin, factor Ⅻa, or factor Ⅺa compared to its unreduced form [91], participating in the endogenous coagulation pathway. LAC activates thrombin by promoting the binding of prothrombin to surface phospholipids and affecting its affinity [92]. 3) Platelet activation: Anti-β2GPi cross-links vWF receptor glycoprotein ibα and ApoE receptor 2 to enhance platelet activation, promote thromboxane A2 (TXA2) release, and increase platelet adhesion [93]. Platelet activation initiates thrombus formation [94]. 4) Complement activation: Complement activation is involved in antiphospholipid antibody-induced thrombosis. Activation of complement by aPL generates C3a and C5a, which mediate leukocyte adhesion and thrombus formation,[95,96]. 5) Inhibition of endothelial nitric oxide synthase (eNOS): By inhibiting eNOS activity and reducing bioavailable NO, aPL promotes leukocyte-endothelial cell adhesion and thrombosis [97]. Autopsy reports of patients with COVID-19 revealed infiltration of leukocytes in small pulmonary vessels; therefore, we hypothesized that aPL also promotes leukocyte adhesion in cerebral vessels [58]. 6) Inhibition of annexin A5: AnnexinV is a calcium-dependent protein that binds to phosphatidylserine residues, which form a shield that inhibits the formation of procoagulant complexes, including the TF-Ⅶa complex, the Ⅸa-Ⅷa complex, and the Ⅹa-Ⅴa complex [98]. The complex of anti-β2GPi and β2GPi can disrupt this shield, exposing procoagulant phosphatidylserines and, hence, promote thrombosis [99].

SARS-CoV-2 triggers the production of aPL by molecular mimicry and increased levels of cytokines[100]. It is reasonable to speculate that aPL serves as a potential mediator of cerebrovascular events in patients with COVID-19.

### Abnormal ferritin metabolism

Ferritin was proven to be a predictor of mortality among 150 COVID-19 patients in Wuhan, since its level is significantly higher in non-survivors than in survivors [101]. Zhou et al. found that COVID-19 patients with elevated serum ferritin levels (>300 µg/L) had a 9-fold increase in pre-discharge mortality [54]. Subsequently, a growing number of researchers have recognized serum ferritin as a powerful index for COVID-19 severity, helping to identify cases with dismal prognosis [102,103]. The first reported case of COVID-19-related stroke showed that the patient’s serum ferritin level was markedly elevated [104], which has attracted researchers’ attention to the role of serum ferritin in COVID-19-related stroke.

Ferritin is an iron-binding molecule that stores major intracellular iron in all organisms [104]. A higher ferritin level is associated with an increased risk of ischemic stroke [105,106]. Ruddell et al. proposed the role of serum ferritin (mainly composed of L-ferritin subunits) as a pro-inflammatory signaling molecule in hepatic stellate cells [107]. Ferritin is involved in inflammatory/fibrotic states associated with the infection of various organs, such as the heart, lung, brain, kidney, and pancreas, all of which have cell types similar to hepatic stellate cells to mediate the fibrotic response to injury [107,108]. Serum ferritin can initiate the production of thrombus-like fibers through a variety of pathways and induce inflammation, thereby damaging cerebral blood vessels and causing neurological symptoms.

## Treatment of COVID-19-related stroke

Multidisciplinary treatment of COVID-19-related stroke, including antiviral drugs, supportive therapy, and stroke treatment, has shown cheerful clinical effects. Alharthy et al. have shown that in life-threatening COVID-19, especially with immune dysregulation features such as antiphospholipid antibodies, therapeutic plasma exchange could be an effective rescue therapy [109]. Similar to stroke alone, treatment for ischemic stroke in COVID-19 patients is individualized and complicated, including thrombolysis [110,111] and thrombectomy [112,113] for acute interventions and antiplatelets and anticoagulants for secondary prevention. It should be noted that many patients with acute ischemic stroke lose the opportunity for acute intervention because of isolation and reluctance to present to the hospital [114,115]. The treatment efficacy for ischemic stroke following COVID-19 needs further evaluation. Ischemic stroke therapy in COVID-19 patients should not only be based on traditional guidelines, but on the experience and new insights from healthcare workers who are combating COVID-19 worldwide [116].

## Conclusions

Despite the characteristic symptoms of respiratory distress, less than 2% of hospitalized patients with COVID-19 have an ischemic stroke [9,14,15,117]. The exact stroke pathophysiology following SARS-CoV-2 infection remains to be established by autopsies and pathology reports. Based on current knowledge, several possible mechanisms exist, including hypercoagulability, activation of the cytokine storm, excessive levels of antiphospholipid antibodies, and abnormal ferritin levels. During the lockdown period, factors such as lifestyle changes and sedentary lifestyle must be taken into consideration when assessing stroke risks [118]. It is unclear whether the patients who recover from COVID-19-related stroke experience any transient or long-lasting cerebrovascular sequelae. Clinical physicians should be aware that severe COVID-19 can lead to ischemic stroke, and close monitoring of the neurological status of COVID-19 patients is imperative.
